# High-Dose α-Tocopherol Supplementation Does Not Induce Bone Loss in Normal Rats

**DOI:** 10.1371/journal.pone.0132059

**Published:** 2015-07-06

**Authors:** Shunji Kasai, Akemi Ito, Kaori Shindo, Tohru Toyoshi, Masahiro Bando

**Affiliations:** 1 Vitamin E Information and Technology Section, Customer Joy Department, Eisai Co. Ltd., Tokyo, Japan; 2 Ito Bone Histomorphometry Institute, Niigata, Japan; 3 Canon Lifecare Solutions Inc., Tokyo, Japan; 4 Study Department, Nihon Bioresearch Inc., Gifu, Japan; 5 Eisai Product Creation Systems, Eisai Co. Ltd., Tsukuba, Japan; Faculté de médecine de Nantes, FRANCE

## Abstract

Oxidative stress affects bone turnover. Preventative effects of antioxidants such as vitamin E on reduced bone mineral density and fractures associated with aging, osteoporosis, and smoking have been examined in animals and humans. The effects of vitamin E (α-tocopherol; αT) on bone health have yielded conflicting and inconclusive results from animal studies. In this study, to determine the bone effects of αT, we investigated the in vivo effects of αT on the bone mineral density, bone mass, bone microstructure, bone resorption, and osteogenesis through peripheral quantitative computed tomography (pQCT) measurements, micro-computed tomography (micro-CT) analyses, and bone histomorphometry of lumbar vertebrae and femurs in normal female Wistar rats fed diets containing αT in different quantities (0, 30, 120, or 600 mg/kg diet) for 8 weeks. To validate our hypotheses regarding bone changes, we examined ovariectomized rats as an osteoporosis model and control sham-operated rats in parallel. As expected, ovariectomized rats had reduced bone mineral density in lumbar vertebrae and the distal metaphyses of their femurs, reduced bone mass and deteriorated microstructure of cancellous bones in the vertebral body and distal femur metaphyses, and reduced bone mass due to resorption-dominant enhanced bone turnover in secondary cancellous bones in these sites. In comparison, αT administered to normal rats, even at the highest dose, did not induce reduced bone mineral density of lumbar vertebrae and femurs or a reduced bone mass or fragile microstructure of cancellous bones of the vertebral body and distal femur metaphyses. Instead, αT-fed rats showed a tendency for an osteogenesis-dominant bone mass increase in secondary cancellous bones in the vertebral body, in which active bone remodeling occurs. Thus, αT consumption may have beneficial effects on bone health.

## Introduction

Osteoporosis is associated with reduced bone strength due to decreased bone mineral density (BMD) and deteriorated bone quality [1]. Osteoporosis is also a major risk factor for fractures. Fractures caused by osteoporosis increase not only mortality rates [2–4] but also care requirements. Secondary skeletal deformities due to fractures are often followed by patients becoming bedridden or developing chronic lumbar spondylosis. These conditions limit their functions of daily living. Preventing osteoporosis and maintaining bone health are key factors that both improve individuals’ quality of life (QOL) and help to resolve serious social problems in an aging society, such as care for the elderly.

Free radicals suppress osteoblast differentiation [5] and facilitate bone resorption by activating osteoclasts [6, 7]. As such, oxidative stress has been suggested to create an imbalance between osteogenesis and bone resorption. For example, a study of middle-aged males (mean age, 55.7 years) and females (mean age, 55.9 years) revealed that the urinary 8-iso-prostaglandin F_2α_ (8-iso-PGF_2α_) level, a biomarker of oxidative stress, was negatively correlated with BMD [8]. A comparative study between female patients ≥ 60 years with post-menopausal osteoporosis (mean age, 70.4 years) and control subjects (mean age, 68.8 years) revealed marked reductions in the levels of blood antioxidants (vitamins C and E and uric acid) and an anti-oxidative enzyme (superoxide dismutase) in the females with osteoporosis [9].

Oxidative stress affects bone turnover. The preventative effects of antioxidants such as vitamin E on reduced BMD and fractures associated with aging, osteoporosis, and smoking have been studied in animals and humans. Most studies using osteoporosis animal models and normal animals [10–14] as well as epidemiological studies in humans [15–20] have supported the beneficial effects of α-tocopherol (αT), a vitamin E homolog. Fujita et al., however, have recently proposed that αT may have adverse effects on bone health in humans, as it reduced the bone mass in mice and rats by promoting osteoclast fusion [21]. However, they did not assess the effects of different αT doses and did not include an appropriate positive control group to ensure the validity of their bone assessments. To determine whether αT has any adverse effects on bones, we used a rat model to study the in vivo effects of αT on various parameters. Using several techniques, we conducted a detailed analysis of BMD, bone mass, bone microstructure, bone resorption, and osteogenesis. Techniques included peripheral quantitative computed tomography (pQCT) measurements, micro-computed tomography (micro-CT) analyses, and bone histomorphometry of the lumbar vertebrae and femurs in normal rats that received different doses of αT. Our results demonstrated that αT did not adversely affect bone health in normal rats and actually suggested that it has beneficial effects.

## Materials and Methods

### Vitamin E

dl-α-tocopheryl acetate (αT) was synthesized by Eisai Co., Ltd. (Tokyo, Japan).

### Diets

Table 1 shows the composition of the αT-deficient diet. αT-supplemented diets were prepared by the addition of 30, 120, or 600 mg/kg of αT to the αT-deficient diet. These diets were purchased from Oriental Yeast Co., Ltd. (Tokyo, Japan).

**Table 1:** Composition of a αT-deficient diet.

Component	Amount (g/kg)
Casein (vitamin-free)	200
DL-methionine	3
Corn starch	430
α-Corn starch	120
Sucrose	100
Corn oil (vitamin E-stripped)	50
Cellulose powder	50
AIN-76 Mineral mixture	35
AIN-76 Vitamin mixture (without αT)	10
Choline bitartrate	2

αT: α-tocopherol

### Animal experiments

Animal experiments were approved by animal experiment committees of Nihon Bioresearch Inc. (Gifu, Japan) and Eisai. All animal treatments and experimental procedures were conducted in accordance with the pertinent institutional regulations.

Five-week-old female Wistar rats were purchased from CLEA Japan (Tokyo, Japan). These rats were allowed to acclimatize to the standard experimental conditions of 23°C–25°C, 47%–70% humidity, and 12-h day and night cycles. The animals had free access to water and the standard rodent diet (CE-2, CLEA Japan). At 6 weeks of age, 16 of 48 rats underwent bilateral ovariectomy (OVX; OVX group, n = 8) or sham surgery (sham; sham group, n = 8) under isoflurane anesthesia. After the surgery, the OVX and sham group rats were fed the αT-deficient diet for 8 weeks and used as the osteoporosis model group and its control counterpart, respectively. The remaining 32 normal rats were randomly divided into 4 groups (n = 8/group), and each group of rats was fed the αT-deficient diet (control group), 30 mg αT/kg diet (low-dose group), 120 mg αT/kg diet (medium-dose group), or 600 mg αT/kg diet (high-dose group) for 8 weeks. These rats had free access to water and diet. Their body weight and food consumption were measured once a week.

Tetracycline hydrochloride (20 mg/kg) (Sigma-Aldrich, St. Louis, MO, USA) and calcein (10 mg/kg) (Sigma-Aldrich) were subcutaneously injected to double-label the bones, 7 days and 2 days before euthanasia, respectively. Blood was collected from the abdominal aorta of rats under anesthesia with isoflurane and centrifuged to obtain the serum. Serum samples were stored at −80°C until analysis. The lumbar vertebrae (L1–L3, L4–L6) and femurs on both sides were resected and fixed with 70% ethanol.

### Measurement of serum αT concentration

After extracting serum samples with hexane, the extracts were dried and dissolved in 20 μl of ethyl acetate and 150 μl of ethanol. αT was measured using high-performance liquid chromatography on a Wakosil-II 5C18 column (4.6 × 250 mm; Wako Pure Chemical Industries, Ltd., Osaka, Japan) using a linear gradient elution system [acetonitrile:dichloromethane:methanol from 85:5:10 to 60:30:10 (v/v)] and a fluorescence detector (excitation, 295 nm; emission, 335 nm).

### Measurement of BMD

BMD (mg/cm^3^) was measured in the L5 lumbar vertebra (the distal one-fourth of the vertebra) and the distal metaphysis (3.0 mm from the growth plate cartilage) and diaphysial center of the left femur by pQCT using an XCT Research SA+ scanner equipped with XCT 6.20 software (Stratec Medizintechnik GmbH, Prorzheim, Germany).

### Micro-CT analysis

The micro-CT system (μCT40; Scano Medical, Bassersdorf, Switzerland) was used for measurement of the bone mass and non-destructive 3-dimensional analysis of the cancellous bones in the L5 lumbar vertebral body and distal metaphysis of the left femur. Three-dimensional analysis was conducted using scanned slice data to compute the following morphometric parameters: trabecular bone volume fraction (BV/TV), trabecular number (Tb.N), trabecular separation (Tb.Sp), connectivity density (Conn.D), and degree of anisotropy (DA).

### Bone histomorphometry

After the removal of soft tissues, the L2 vertebra and the distal part of the right femur were subjected to Villanueva bone staining for 7 days without decalcifying treatment [22], dehydrated with increasing concentrations of ethanol, and embedded in methyl methacrylate (Wako Pure Chemical Industries, Ltd.). Sagittal sections of the vertebral body (5-μm thick) and slices of the frontal section of the distal femur (5-μm thick) were prepared using a microtome (LIECA, Germany).

In vertebral body measurements, regions of the secondary cancellous bone located at least 939 μm away from the growth plate cartilage and at least 313 μm away from the endosteum surface of the cortical bone were selected on both sides. The measured area was 2.45 mm^2^. In femur measurements, regions of the secondary cancellous bone located 626–1565 μm proximal to the growth plate cartilage of the distal femur were selected. The measured area was 2.35 mm^2^. The nomenclature and units used were in accordance with the ASBMR Histomorphometry Nomenclature Committee [23]. The following parameters were measured: bone volume/tissue volume (BV/TV), eroded surface/bone surface (ES/BS), osteoid volume/osteoid surface (OV/OS), osteoid thickness (O.Th), mineral apposition rate (MAR), number of osteoclast/bone surface (N.Oc/BS), and number of osteoblast/bone surface (N.Ob/BS). N.Oc was separately measured for mononuclear osteoclast (N.Mo.Oc) and multinuclear osteoclast (N.Mu.Oc).

### Statistical analysis

All the data are shown as the mean value ± standard error of the mean (SEM). SAS version 8.2 (SAS Institute; Tokyo, Japan) was used for statistical analysis. The significant difference was determined by using independent Student’s t-test (sham group vs. OVX group) or Dunnett’s multiple comparison test (αT-deficient diet-fed normal rat control group vs. αT-supplemented diet-fed normal rat group). A P-value (both sides) of <0.05 was considered statistically significant.

## Results

### Body weight, food intake, and αT intake

αT supplementation had no significant effect on the body weight in normal rats (Table 2), while OVX rats showed a significant increase in body weight (P < 0.001). No significant differences in the mean food intake (g/kg body weight/day) were observed between the sham and OVX groups or between the control and αT-supplemented groups of normal rats over the course of the study period (Table 2). Based on the mean food intake and αT content in the diet, we determined the mean αT intake during the study period. The mean αT intake levels during the study period in the low-, medium-, and high-dose groups of rats were 2.2 ± 0.0, 9.3 ± 0.1, and 45.1 ± 0.9 mg/kg body weight/day, respectively.

**Table 2:** Effects of αT supplementation on body weight, food intake, and serum αT levels.

Groups	Body weight (g)		Average daily food intake (g/kg body weight/day)	Serum αT level (μg/dL)
	initial	final		
Sham rats	136±2	253±4	80±1	59±4
OVX rats	136±1	328±4***	78±2	50±2
Normal rats				
Control	136±1	253±7	78±1	54±2
Low-dose αT	136±1	252±7	75±1	618±48^†^
Medium-dose αT	136±2	256±5	78±1	1050±91^†††^
High-dose αT	136±1	259±5	75±1	2163±256^†††^

OVX: ovariectomy; αT: α-tocopherol

Values are mean ± standard error of the mean.

***P < 0.001 vs. sham group (Student’s t-test).

^†^P < 0.05

^†††^P < 0.001 vs. control group (Dunnett’s multiple comparison test).

### Effects of αT supplementation on serum αT level

The serum αT levels in normal rats that received low-, medium-, and high-dose supplementation of αT were 11-fold (P < 0.05), 19-fold (P < 0.001), and 40-fold (P < 0.001) higher, respectively, with statistical significance in comparison with the serum αT level in control rats (Table 2).

### Effects of αT supplementation on BMD

**Fig 1:**
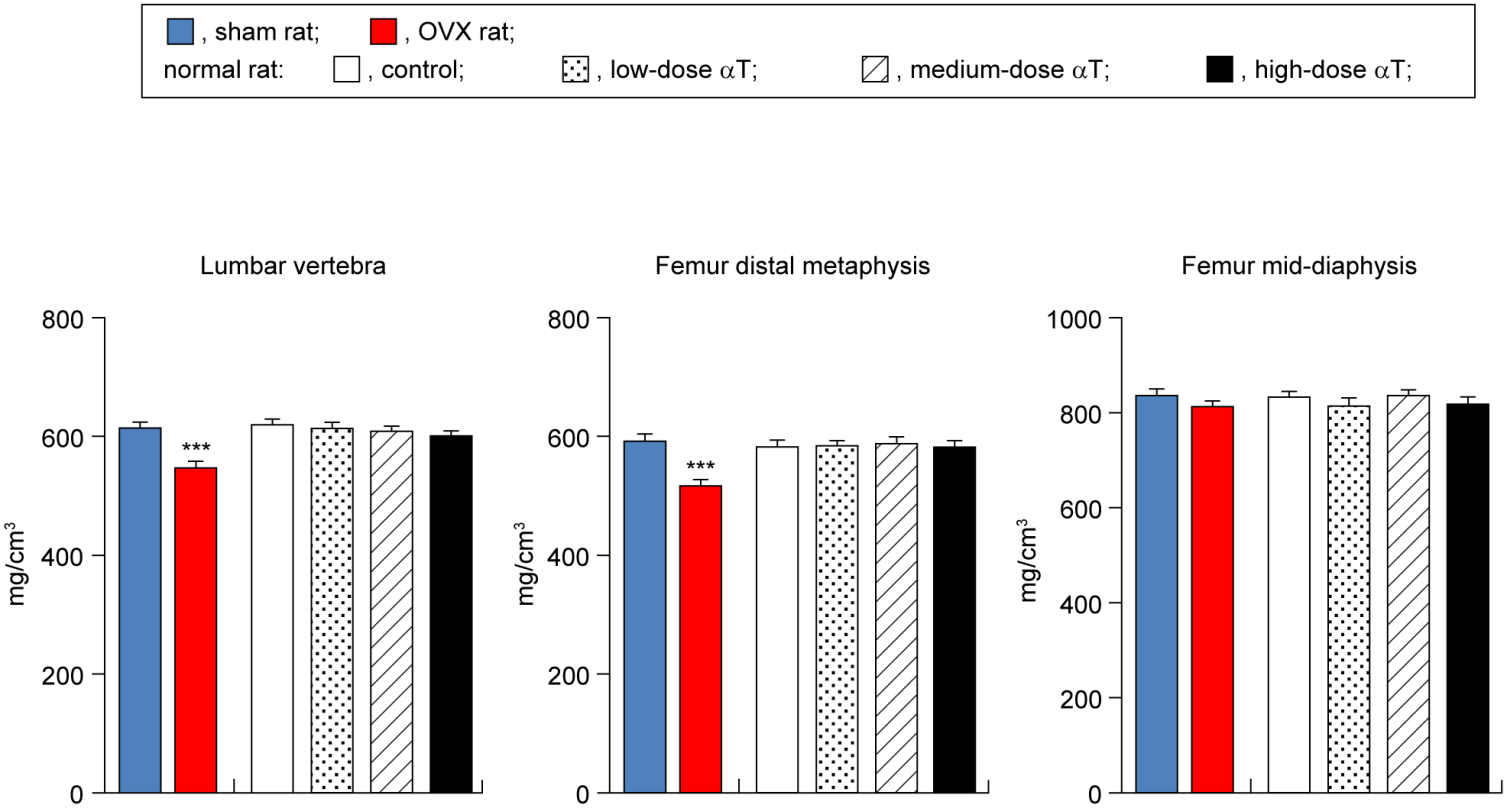
αT supplementation effects on bone mineral density of lumbar vertebra and femur in normal rats. Data are presented as mean ± standard error of the mean. ***P < 0.001 vs. sham rat group.

Bone mineral densities in the lumbar vertebra and the distal metaphysis of the femur, which are rich in cancellous bones, in OVX rats were 11% (P < 0.001) and 13% (P < 0.001) lower, respectively, with statistical significance in comparison with those in sham rats. However, there were no significant differences between these groups of rats in terms of BMD of the distal femur diaphysis, which is rich in the cortical bone (Fig 1).

### Effects of αT supplementation on bone mass and microstructure of cancellous bone

The effects of OVX treatment and αT supplementation on the bone mass and microstructure were assessed in the vertebral body and distal femur diaphysis by micro-CT analysis.

**Fig 2:**
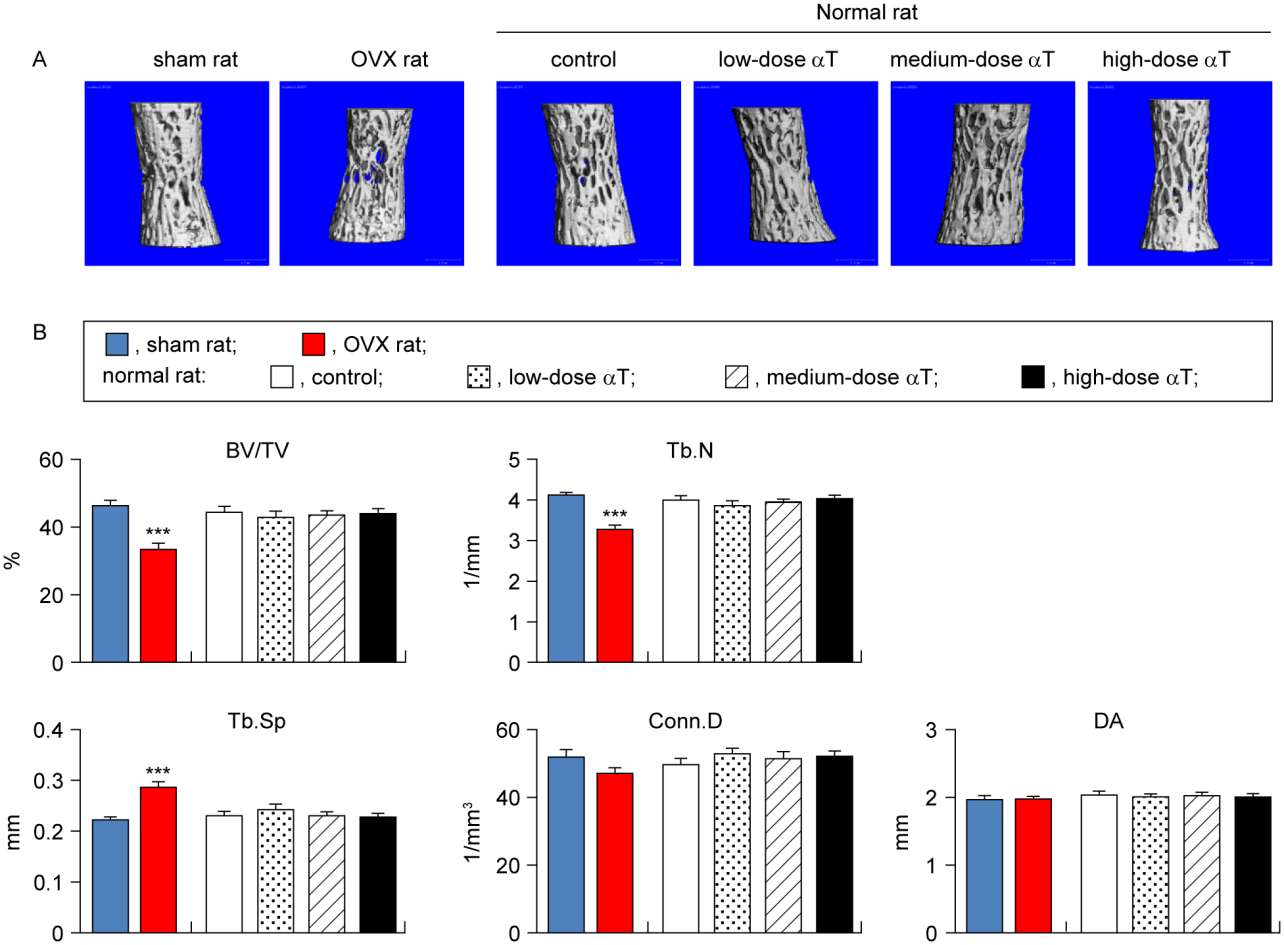
Micro-CT analysis of trabecular bone in vertebral body. (A) Representative 3-dimensional micro-CT images of trabecular bones in vertebral bodies are shown. (B) Microstructural parameters (BV/TV, trabecular bone volume fraction; Tb.N, trabecular number; Tb.SP, trabecular separation; Conn.D, connectivity density; DA, degree of anisotriphy) of trabecular bones in vertebral bodies are shown. Data are presented as mean ± standard error of the mean. ***P < 0.001 vs. sham rat group.

**Fig 3:**
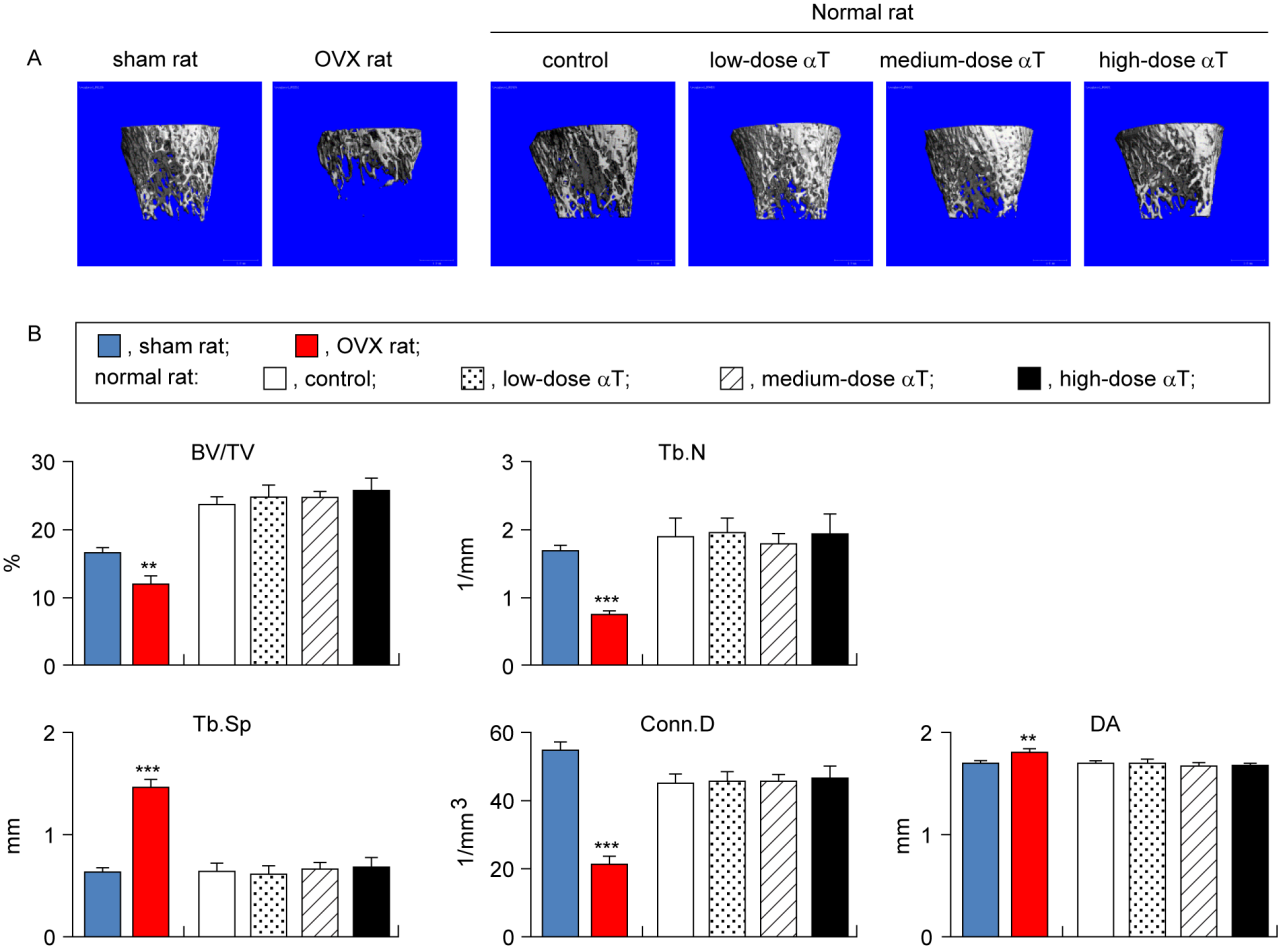
Micro-CT analysis of trabecular bones in distal femur metaphysis. (A) Representative 3-dimensional micro-CT images of trabecular bones in distal femur metaphysis are shown. (B) Microstructural parameters (BV/TV, trabecular bone volume fraction; Tb.N, trabecular number; Tb.SP, trabecular separation; Conn.D, connectivity density; DA, degree of anisotriphy) of trabecular bones in distal femur metaphysis are shown. Data are presented as mean ± standard error of the mean. **P < 0.01, ***P < 0.001 vs. sham rat group.

For the vertebral cancellous bone, OVX rats showed a 28% decreased BV/TV (P < 0.001), 20% decreased Tb.N (P < 0.001), and 29% increased Tb.Sp (P < 0.001) in comparison with sham rats. For the distal femur diaphysis, OVX rats showed a 28% decreased BV/TV (P < 0.01), 55% decreased Tb.N (P < 0.001), 128% increased Tb.Sp (P < 0.001), 61% decreased Conn.D (P < 0.001), and 6% increased DA (P < 0.01) in comparison with sham rats (Figs 2 and 3). Thus, compared with sham rats, OVX rats were confirmed to have a reduced cancellous bone mass and weakened microstructures.

αT supplementation in normal rats had no significant effects on the bone mass or microstructure parameters of cancellous bones in the vertebral body and distal femur metaphysis (Figs 2 and 3).

### Effects of αT supplementation on secondary cancellous bone

To assess the effects of OVX treatment and αT supplementation in normal rats on bone turnover, we conducted bone histomorphometry with secondary cancellous bones in the vertebral body and distal femur metaphysis, which have high bone turnover [24].

With regard to the secondary cancellous bone in the vertebral body, a significant 22% reduction in BV/TV (P < 0.01) was found in OVX rats in comparison with sham rats; this was accompanied by a 29% increase in the bone resorption parameter ES/BS (P < 0.05) and a 50% increase in OV/OS (P < 0.001), 50% increase in O.Th (P < 0.001), 31% increase in MAR (P < 0.001), and 133% increase in N.Ob/BS (P < 0.001) as osteogenesis parameters. Similarly, with regard to the secondary cancellous bone of the distal femur metaphysis, OVX rats showed a significant 52% reduction in BV/TV (P < 0.001) in comparison with sham rats. This reduction was accompanied by an 18% increase in ES/BS (P = 0.208), a 29% increase in OV/OS (P < 0.001), a 28% increase in O.Th (P < 0.001), a 36% increase in MAR (P < 0.001), and a 108% increase in N.Ob/BS (P < 0.001). These data confirmed that the bone mass was reduced in OVX rats as a result of enhanced bone resorption and osteogenesis in secondary cancellous bones in the vertebral body and distal femur metaphysis.

**Fig 4:**
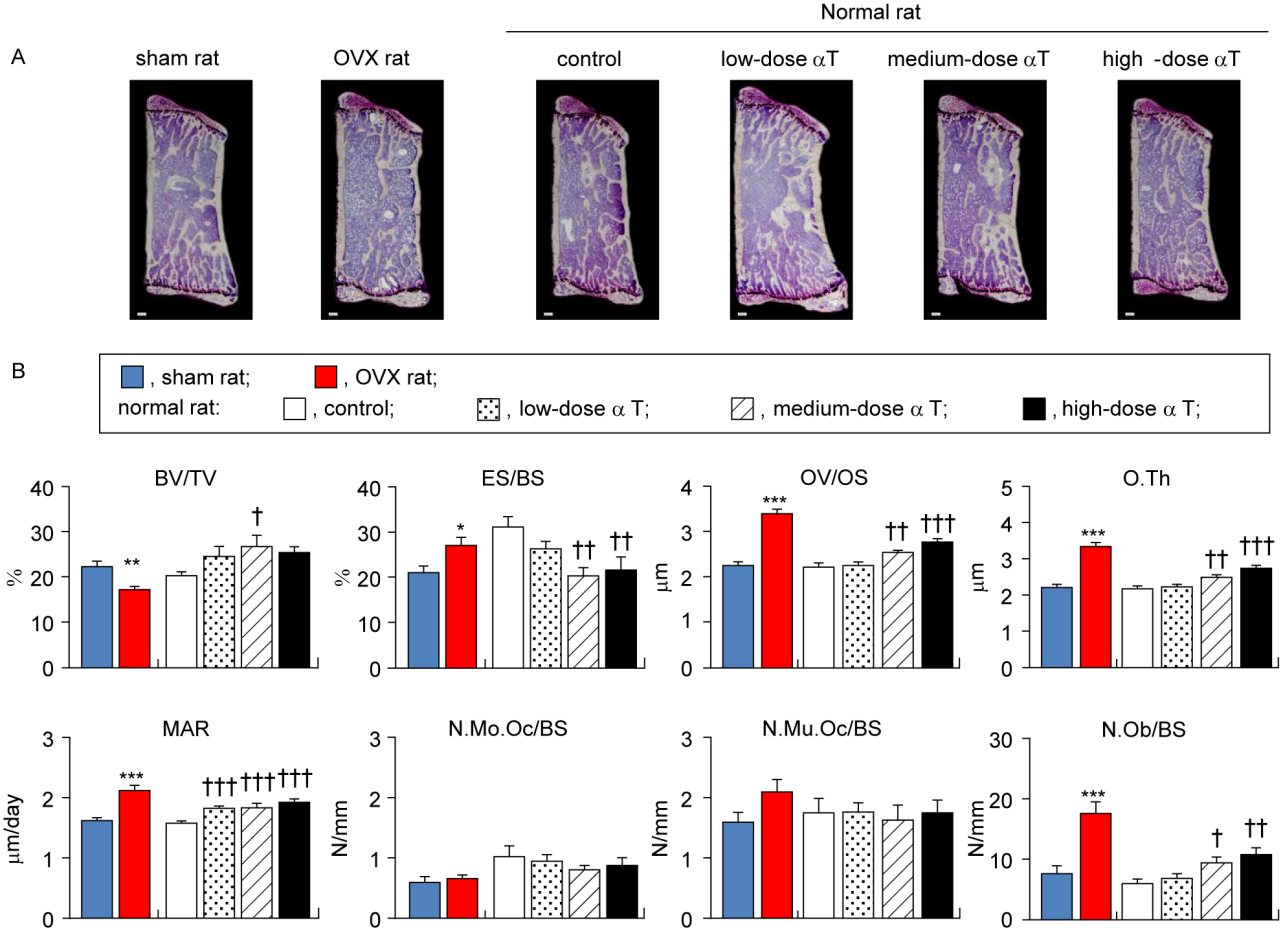
Histomorphometric analysis of secondary cancellous bones in vertebral body. (A) Representative micrographs of sagittal sections of vertebral bodies stained with Villanueva bone staining without decalcifying treatment are shown. (B) Histomorphometrical parameters (BV/TV, bone volume/tissue volume; ES/BS, eroded surface/bone surface; OV/OS, osteoid volume/osteoid surface; O.Th, osteoid thickness; MAR, mineral apposition rate; N.Mo.Oc/BS, number of mononuclear osteoclast/bone surface; N.Mu.Oc/BS, number of multinuclear osteoclast/bone surface; and N.Ob/BS, number of osteoblast/bone surface) of secondary spongiosa in vertebral bodies are shown. Data are presented as mean ± standard error of the mean. *P < 0.05, **P < 0.01, and ***P < 0.001 vs. sham rat group; ^†^P < 0.05, ^††^P < 0.01, and ^†††^P < 0.001 vs. normal rat control group.

**Fig 5:**
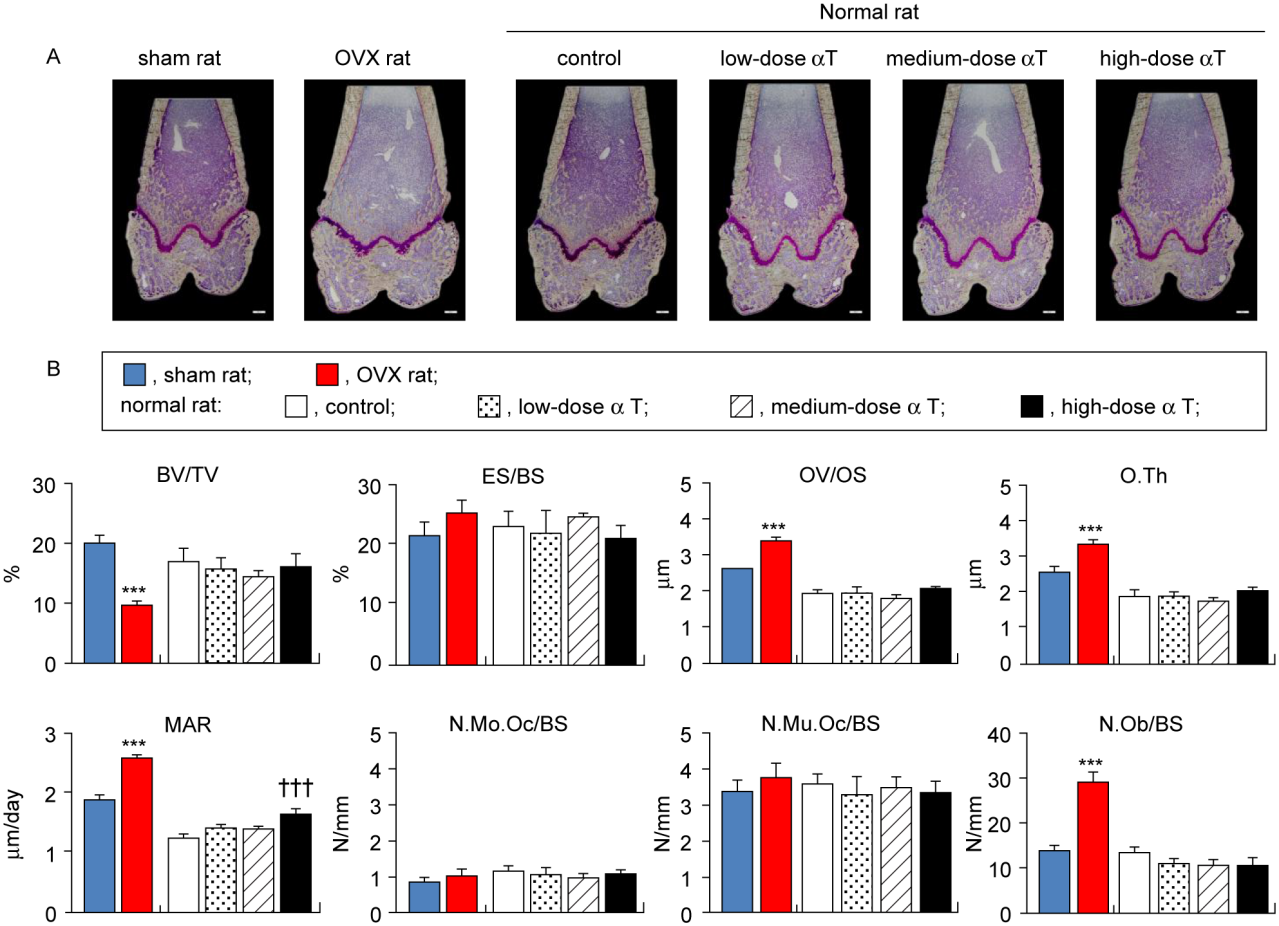
Histomorphometric analysis of secondary cancellous bones in distal femur metaphysis. (A) Representative micrographs of slices of frontal section of distal femur stained with Villanueva bone staining without decalcifying treatment are shown. (B) Histomorphometrical parameters (BV/TV, bone volume/tissue volume; ES/BS, eroded surface/bone surface; OV/OS, osteoid volume/osteoid surface; O.Th, osteoid thickness; MAR, mineral apposition rate; N.Mo.Oc/BS, number of mononuclear osteoclast/bone surface; N.Mu.Oc/BS, number of multinuclear osteoclast/bone surface; and N.Ob/BS, number of osteoblast/bone surface) of secondary cancellous bones in distal femur metaphysis are shown. Data are presented as mean ± standard error of the mean. ***P < 0.001 vs. sham rat group; ^†††^P < 0.001 vs. normal rat control group.

αT supplementation had different effects on the bone turnover of secondary cancellous bones in the vertebral body and distal femur metaphysis in normal rats. For the vertebral secondary cancellous bone, αT-supplemented normal rats had a significant increase or a trend for increase in BV/TV in comparison with control rats [low dose, +21% (P = 0.189); medium dose, +32% (P < 0.05); and high dose, +25% (P = 0.099)], which was accompanied by reduced ES/BS [medium dose, −34% (P < 0.01); high dose, −30% (P < 0.01)], increased OV/OS [medium dose, +14% (P < 0.01); high dose, +24% (P < 0.001)], increased O.Th [medium dose, +15% (P < 0.01); high dose, +25% (P < 0.001)], and increased MAR [low dose, +16% (P < 0.001); medium dose, +17% (P < 0.001); high dose, +22% (P < 0.001)]. Although the number of osteoblasts was significantly higher in αT-supplemented normal rats than in control rats [medium dose, +59% (P < 0.05); high dose, +82% (P < 0.01)], no significant changes were found in the number of osteoclasts, in the mononuclear subpopulation (N.Mo.Oc/BS) or the multinuclear subpopulation [(N.Mu.Oc/BS); Fig 4]. In contrast to the secondary cancellous bone in the vertebral body, αT supplementation did not significantly alter any of the osteogenic or bone resorption parameters in bone histomorphometry for the secondary cancellous bone of the distal femur metaphysis, except for MAR (Fig 5). These results indicate that αT supplementation in normal rats resulted in a trend for increased bone mass in the vertebral secondary cancellous bone through reducing bone resorption and enhancing osteogenesis but had virtually no effect on the secondary cancellous bone in the distal femur metaphysis.

## Discussion

In the present study, we assessed the effects of αT on bones in normal rats fed diets containing different levels of αT. We found that αT did not induce BMD reduction in the lumbar vertebrae or distal femur metaphysis even at the highest dose tested; it did not induce bone mass reduction and microstructure deterioration in the cancellous bones in the vertebral body or distal femur metaphysis. Rather, αT supplementation resulted in a tendency for an osteogenesis-dominant bone mass increase in the vertebral secondary cancellous bone, in which active bone remodeling occurs.

OVX rats are widely used in animal models of osteoporosis. This is a model that offers high reproducibility. It can be characterized by high bone turnover resulting from enhanced bone resorption and osteogenesis due to estrogen deficiency and reduced bone mass because of the bone resorption rate exceeding the osteogenesis rate [24]. To ensure that we correctly assessed bone changes, we performed assessments with the osteoporosis model rats and control sham-operated rats. In agreement with previous studies [25–34], we successfully demonstrated several points: 1) weight gain (Table 2), 2) reduced BMD in the lumbar vertebra and distal metaphysis of the femur (Fig 1), 3) reduced bone mass and deteriorated microstructure in the cancellous bones of the vertebral body and distal femur metaphysis (Figs 2 and 3), and 4) reduced bone mass due to bone resorption-dominant enhanced bone turnover in the secondary cancellous bones of the vertebral body and distal femur metaphysis (Figs 4 and 5). All these indicated that our assessments were valid. In addition to OVX rats, we evaluated the bones of normal rats fed diets containing different levels of αT and found the following characteristics of the effects of αT on normal rat bones: First, αT administered to normal rats, even at the highest dose, did not reduce BMD in the lumbar vertebra or femur (Fig 1). It also did not reduce the bone mass or induce microstructure deterioration of the cancellous bone in the vertebral body or distal metaphysis of the femur (Figs 2 and 3). Second, αT supplementation resulted in a tendency for increased bone mass in the secondary cancellous bone in the vertebral body in normal rats; however, it had no effect on the secondary cancellous bone in the distal femur metaphysis (Figs 4 and 5). In the present study, rats were sacrificed at 14 weeks of age. At this age, remodeling dominates bone turnover in the cancellous bone of the vertebral body, whereas modeling is dominant in the cancellous bones in distal metaphyses of the long bones [35–37]. Based on these findings, the vertebral body-selective effects of αT may be due to the specificity of αT effects to remodeling. The third feature of αT effects on the bones of normal rats was that an αT-induced tendency for increased bone mass in the vertebral secondary cancellous bone involved the suppression of bone resorption and promotion of osteogenesis (Fig 4).

With regard to the effects of αT on bone health, conflicting results have been reported for animal experiments. Most of these reports supported the beneficial effects of αT on bone health. Hermizi et al. [10] reported that in a nicotine-induced osteoporosis rat model, αT ameliorated the nicotine-induced bone mass reduction, bone resorption enhancement, and osteogenesis suppression in the femur. Norazlina et al. [11] reported that in OVX rats, αT suppressed BMD decrease in the femur and lumbar vertebra associated with OVX. Similarly, Muhammad et al. reported that αT improved OVX-associated bone mass decrease and bone microstructure deterioration in this osteoporosis model rats [12]. In addition, Muhammad et al. reported that αT suppressed bone resorption and facilitated osteogenesis in normal rats and increased bone strength [13, 14]. In contrast to these results, Fujita et al. recently proposed a hypothesis that αT reduces bone mass via the facilitation of osteoclast fusion and thereby exerts an adverse effect on bone health [21]. Their hypothesis was based on 3 experimental results: 1) When normal mice and rats in their growth periods were fed diets containing αT at 600 mg/kg diet for 8 weeks, their bone mass decreased by 20% compared with that of animals fed normal diets; this was accompanied by enhanced bone resorption and enlarged osteoclasts. 2) The bone mass of mice with low αT levels in their bodies due to deletion of the αT transfer protein (αTTP) was higher than that of wild-type mice; αT supplementation in these αTTP knockout mice reduced the bone mass. 3) In vitro experiments demonstrated that αT facilitated osteoclast fusion and formation of multinuclear osteoclasts, which have a high bone resorption capability. One of our aims in the present study was to assess the hypothesis proposed by Fujita et al. using normal rats. Our study with normal rats in their growth period was conducted under the same conditions as those used by Fujita et al., including the strain and age in weeks of the rats, αT contents in the diet, and feeding period. The only exception was the gender of the rats. In the present study, young normal rats that received αT-supplemented diet did not show an increased number of multinuclear osteoclasts, reduced cancellous bone mass, deteriorated cancellous bone microstructure, or enhanced bone resorption. Thus, our experimental results did not support the hypothesis proposed by Fujita et al. Recently, Iwaniec et al. also assessed the hypothesis of Fujita et al. using normal adult rats with mature skeletons and old αTTP knockout rats [38]. They reported that their results also did not support the hypothesis.

In the present study, the mean αT intake levels in normal rats in the low-, medium-, and high-dose groups were 2.2, 9.3, and 45.1 mg/kg body weight/day, respectively, which are equivalent to 132, 558, and 2,708 mg/day, respectively, in a 60-kg human. The highest dose used in the present study exceeded 1,000 mg/day [39]. This is the tolerable upper level of regular daily vitamin E intake for an adult that is likely to pose no risk of adverse health effects for most people in the general population, as established by The Food and Nutrition Board (FNB) of the Institute of Medicine, National Academies (United States). Thus, the results of the present study suggest that αT consumption by humans below the tolerable upper intake level is unlikely to adversely affect bones.

The present study had some limitations. We used young female rats that received αT for 8 weeks. Further studies should be conducted in rats with a different gender, ages, and duration of αT administration. In addition, in the present study, we noted that αT tended to increase the secondary cancellous bone mass in the vertebral body, in association with suppressed bone resorption and enhanced osteogenesis; however, the underlying mechanism remains to be determined. This mechanism should be elucidated at the molecular level in the future.

In conclusion, αT supplementation was demonstrated to cause no adverse effects on the bone at a high dose but tended to induce an osteogenesis-dominant bone mass increase in the vertebral secondary cancellous bone, in which active bone remodeling occurs. Therefore, αT consumption may have beneficial effects on bone health.

## References

[1] NIH Consensus Development Panel on Osteoporosis Prevention, Diagnosis, and Therapy (2001) Osteoporosis prevention, diagnosis, and therapy. JAMA 285: 785–795. 11176917

[2] Ensrund KE, Thompson DE, Cauley JA, Nevitt MC, Kado DM, Hochberg MC, et al (2000) Prevalent vertebral deformities predict mortality and hospitalization in older women with low bone mass. Fracture Intervention Trial Research Group. J Am Geriatr Soc 48: 241–249. 10733048 10.1111/j.1532-5415.2000.tb02641.x

[3] Nguyen ND, Center JR, Eisman JA, Nguyen TV (2007) Bone loss, weight loss, and weight fluctuation predict mortality risk in elderly men and women. J Bone Miner Res 22: 1147–1154. 17635040 10.1359/jbmr.070412

[4] Suzuki T, Yoshida H (2010) Low bone mineral density at femoral neck is a predictor of increased mortality in elderly Japanese women. Osteoporos Int 21: 71–79. 10.1007/s00198-009-0970-6 19499274

[5] Mody N, Parhami F, Sarafian TA, Demer LL (2001) Oxidative stress modulates osteoblastic differentiation of vascular and bone cells. Free Radic Biol Med 31: 509–519. 11498284 10.1016/s0891-5849(01)00610-4

[6] Garrett IR, Boyce BF, Oreffo RO, Bonewald L, Poser J, Mundy GR. (1990) Oxygen-derived free radicals stimulate osteoclastic bone resorption in rodent bone in vitro and in vivo. J Clin Invest 85: 632–639. 2312718 10.1172/JCI114485 PMC296476

[7] Suda N, Morita I, Kuroda T, Murota S (1993) Participitation of oxidative stress in the process of osteoclast differentiation. Biochim Biophys Acta 1157:318–323. 8323962 10.1016/0304-4165(93)90116-p

[8] Basu S, Michaëlsson K, Olofsson H, Johansson S, Melhus H (2001) Association between oxidative stress and bone mineral density. Boichem Biophys Res Commun 288:275–279. 10.1006/bbrc.2001.5747 11594785

[9] Maggio D, Barabani M, Pierandrei M, Polidori MC, Catani M, Mecocci P, et al (2003) Marked decrease in plasma antioxidants in aged osteoporotic women: results of a cross-sectional study. J Clin Endocrinol Metab 88: 1523–1527. 12679433 10.1210/jc.2002-021496

[10] Hermizi H, Faizah O, Ima-Nirwana S, Ahmad Nazrun S, Norazlina M (2009) Beneficial effects of tocotrienol and tocopherol on bone histomorphometric parameters in sprague-dawley male rats after nicotine cessation. Calcif Tissue Int 84: 65–74. 10.1007/s00223-008-9190-x 19020790

[11] Norazlina M, Ima-Nirwana S, Gapor MT, Khalid BA (2000) Palm vitamin E is comparable to alpha-tocopherol in maintaining bone mineral density in ovariectomised female rats. Exp Clin Endocrinol Diabetes 108: 305–310. 10961363 10.1055/s-2000-7758

[12] Muhammad N, Luke DA, Shuid AN, Mohamed N, Soelaiman IN (2012) Two different isomers of vitamin e prevent bone loss in postmenopausal osteoporosis rat model. Evid Based Complement Alternat Med 2012:161527 10.1155/2012/161527 23118785 PMC3484319

[13] Mehat MZ, Shuid AN, Mohamed N, Muhammad N, Soelaiman IN (2010) Beneficial effects of vitamin E isomer supplementation on static and dynamic bone histomorphometry prameters in normal male rats. J Bone Miner Metab 28: 503–509. 10.1007/s00774-010-0159-2 20145960

[14] Shuid AN, Mehat Z, Mohamed N, Muhammad N, Soelaiman IN (2010) Vitamin E exhibits bone anabolic actions in normal male rats. J Bone Miner Metab 28: 149–156. 10.1007/s00774-009-0122-2 19779668

[15] Östman B, Michaëlsson K, Byberg L, Helmersson J, Basu S, Melhus H, et al (2009) Oxidative stress and bone mineral density in elderly men: antioxidant activity of alpha-tocopherol. Free Radic Biol Med 47: 668–673. 10.1016/j.freeradbiomed.2009.05.031 19500667

[16] Zhang J, Munger RG, West NA, Cutler DR, Wengreen HJ, Corcoran CD, et al (2006) Antioxidant intake and risk of osteoporotic hip fracture in Utah: an effect modified by smoking status. Am J Epidemiol 163: 9–17. 16306312 10.1093/aje/kwj005

[17] Melhus H, Michaëlsson K, Holmberg L, Wolk A, Ljunghall S (1999) Smoking, antioxidant vitamins, and the risk of hip fracture. J Bone Miner Res 14: 129–135. 9893075 10.1359/jbmr.1999.14.1.129

[18] Mata-Granados JM, Cuenca-Acebedo R, Luque de Castro MD, Quesada Gómez JM (2013) Lower vitamin E serum levels are associated with osteoporosis in early postmenopausal women: a cross-sectional study. J Bone Miner Metab 31: 455–460. 10.1007/s00774-013-0432-2 23536191

[19] Michaëlsson K, Wolk A, Byberg L, Ärnlöv J, Melhus H (2014) Intake and serum concentrations of α-tocopherol in relation to fractures in elderly women and men: 2 cohort studies. Am J Clin Nutr 99:107–114. 10.3945/ajcn.113.064691 24225359 PMC3862449

[20] Holvik K, Gjesdal CG, Tell GS, Grimnes G, Schei B, Apalset EM, et al (2014) Low serum concentrations of alpha-tocopherol are associated with increased risk of hip fracture. A NOREPOS study. Osteoporos Int 25:2545–2554. 10.1007/s00198-014-2802-6 25062727

[21] Fujita K, Iwasaki M, Ochi H, Fukuda T, Ma C Miyamoto T, et al (2012) Vitamin E decreases bone mass by stimulating osteoclast fusion. Nat Med 18:589–594. 10.1038/nm.2659 22388090

[22] Villanueva AR, Lundin KD (1989) A versatile new mineralized bone stain for simultaneous assessment of tetracyclin and osteoid seams. Stain Technol 64: 129–138. 2480003 10.3109/10520298909106985

[23] Parfitt AM, Drezner MK, Glorieux FH, Kanis JA, Malluche H, Meunier PJ, et al (1987) Bone histomorphometry: standardization of nomenclature, symbols, and units. Report of the ASBMR Histomorphometry Nomenclature Committee. J Bone Miner Res 2: 595–610. 3455637 10.1002/jbmr.5650020617

[24] Turner RT, Vandersteenhoven JJ, Bell NH (1987) The effects of ovariectomy and 17β-estradiol on cortical bone histomorphometry in growing rats. J Bone Miner Res 2: 115–122. 3455160 10.1002/jbmr.5650020206

[25] Wronski TJ, Cintrón M, Dann LM (1988) Temporal relationship between bone loss and increased bone turnover in ovariectomized rats. Calcif Tissue Int 43: 179–183. 3141020 10.1007/BF02571317

[26] Wronski TJ, Dann LM, Scott KS, Cintrón M (1989) Long-term effects of ovariectomy and aging on the rat skeleton. Calcif Tissue Int 45: 360–366. 2509027 10.1007/BF02556007

[27] Wronski TJ, Dann LM, Horner SL (1989) Time course of vertebral osteopenia in ovariectomized rats. Bone 10: 295–301. 2803866 10.1016/8756-3282(89)90067-7

[28] Wronski TJ, Yen CF (1992) The ovariectomized rat as an animal model for postmenopausal bone loss. Cells Mater Suppl 1: 69–74.

[29] Kalu DN (1991) The ovariectomized rat model of postmenopausal bone loss. Bone Miner 15: 175–191. 1773131 10.1016/0169-6009(91)90124-i

[30] Seedor JG, Quartuccio HA, Thompson DD (1991) The bisphosphonate alendronate (MK-217) inhibits bone loss due to ovariectomy in rats. J Bone Miner Res 6:339–346. 1858520 10.1002/jbmr.5650060405

[31] Wronski TJ, Walsh CC, Ignaszewski LA (1986) Histologic evidence for osteopenia and increased bone turnover in ovariectomized rats. Bone 7: 119–123. 3718786 10.1016/8756-3282(86)90683-6

[32] Black D, Farquharson C, Robins SP (1989) Excretion of pyridinium cross-links of collagen in ovariectomized rats as urinary markers for increased bone resorption. Calcif Tissue Int 44:343–347. 2496906 10.1007/BF02556315

[33] Shiraishi A, Higasi S, Masaki T, Saito M, Ito M, Ikeda S, et al (2002) A comparison of alfacalcidol and menatetrenone for the treatment of bone loss in an ovariectomized rat model of osteoporosis. Calcif Tissue Int 71: 69–79. 12073154 10.1007/s00223-001-2090-y

[34] Otomo H, Sakai A, Ikeda S, Tanaka S, Ito M (2004) Regulation of mineral-to-matrix ratio of lumbar trabecular bone in ovariectomized rats treated with risedronate in combination with or without vitamin K2. J Bone Miner Metab 22: 404–414. 15316861 10.1007/s00774-004-0502-6

[35] Erben RG (1996) Trabecular and endocortical bone surfaces in the rat: modeling or remodeling? Anat Rec 246:39–46. 8876822 10.1002/(SICI)1097-0185(199609)246:1<39::AID-AR5>3.0.CO;2-A

[36] Iwaniec UT, Turner RT (2008) Animal models of osteoporosis In: Marcus R, Feldman D, Nelson DA, Rosen CJ, editors. Osteoporosis, 3rd ed Amsterdam: Elsevier pp 985–1110.

[37] Lelovas PP, Xanthos TT, Thoma SE, Lyritis GP, Dontas IA (2008) The laboratory rat as an animal model for osteoporosis research. Comp Med 58:424–430. 19004367 PMC2707131

[38] Iwaniec UT, Turner RT, Smith BJ, Stoecker BJ, Rust A, Zhang B, et al (2013) Evaluation of long-term vitamin E insufficiency or excess on bone mass, density, and microarchitecture in rodents. Free Radic Biol Med 65: 1209–1214. 10.1016/j.freeradbiomed.2013.09.004 24051180 PMC3859709

[39] Food and Nutrition Board, Institute of Medicine (2000) Dietary reference intakes for vitamin C, vitamin E, selenium, and carotenoids A report of the Panel on Dietary Antioxidants and Related Compounds, Subcommittees on Upper Reference Levels of Nutrients and Interpretation and Uses of Dietary Reference Intakes, and the Standing Committee on the Scientific Evaluation of Dietary Reference Intakes. Washington, DC: National Academy Press.

